# Phytochemical Profile, Toxicological Screening, Antitumor Activity, and Immunomodulatory Response of Saline Extract from *Euphorbia hirta* L. Leaves

**DOI:** 10.3390/molecules30153105

**Published:** 2025-07-24

**Authors:** Jainaldo Alves da Costa, Amanda de Oliveira Marinho, Robson Raion de Vasconcelos Alves, Matheus Cavalcanti de Barros, Isabella Coimbra Vila Nova, Sheilla Andrade de Oliveira, João Victor de Oliveira Alves, Vitória Figueiredo Silva, Magda Rhayanny Assunção Ferreira, Alisson Macário de Oliveira, Luiz Alberto Lira Soares, Carina Scanoni Maia, Fernanda das Chagas Ângelo Mendes Tenório, Virgínia Maria Barros de Lorena, Roberto Araújo Sá, Thiago Henrique Napoleão, Leydianne Leite de Siqueira Patriota, Maria Lígia Rodrigues Macedo, Patrícia Maria Guedes Paiva

**Affiliations:** 1 Departamento de Bioquímica, Universidade Federal de Pernambuco, Recife 50670-901, Pernambuco, Brazil; jainaldo.costa@ufpe.br (J.A.d.C.); amanda.marinho@ufpe.br (A.d.O.M.); robson.raion@ufpe.br (R.R.d.V.A.); matheus.cavalcantibarros@ufpe.br (M.C.d.B.); isabella.coimbra@ufpe.br (I.C.V.N.); joao.oliveiraalves@ufpe.br (J.V.d.O.A.); alisson.macario@ufpe.br (A.M.d.O.); thiago.napoleao@ufpe.br (T.H.N.); leydianne.patriota@ufpe.br (L.L.d.S.P.); 2 Instituto Aggeu Magalhães, Fundação Oswaldo Cruz, Recife 50740-465, Pernambuco, Brazil; sheilla.andrade@fiocruz.br (S.A.d.O.); virginia.lorena@fiocruz.br (V.M.B.d.L.); 3 Departamento de Tecnologia de Alimentos e da Saúde, Faculdade de Ciências Farmacêuticas, Alimentos e Nutrição, Universidade Federal do Mato Grosso do Sul, Campo Grande 79070-900, Mato Grosso do Sul, Brazil; vitoriaf.alunoufms@yahoo.com (V.F.S.); ligia.macedo@ufms.br (M.L.R.M.); 4 Departamento de Engenharia Biomédica, Universidade Federal de Pernambuco, Recife 50670-901, Pernambuco, Brazil; magda.raferreira@ufpe.br; 5 Departamento de Farmácia, Universidade Federal de Pernambuco, Recife 50670-901, Pernambuco, Brazil; luiz.albertosoares@ufpe.br; 6 Departamento de Histologia e Embriologia, Universidade Federal de Pernambuco, Recife 50670-901, Pernambuco, Brazil; carina.maia@ufpe.br (C.S.M.); fernanda.angelo@ufpe.br (F.d.C.Â.M.T.); 7 Centro Acadêmico do Agreste, Universidade Federal de Pernambuco, Caruaru 55014-215, Pernambuco, Brazil; roberto.asa@ufpe.br

**Keywords:** acute toxicity, genotoxicity, antitumor, phenolic compounds

## Abstract

*Euphorbia hirta* L. is traditionally used to treat tumors and has demonstrated anticancer effects. This study evaluated the phytochemical composition, toxicity, and antitumor activity of saline extract (SE) from *E. hirta* leaves in mice. Phytochemical analysis included thin layer chromatography, high-performance liquid chromatography, and quantification of phenols, flavonoids, and proteins. Acute toxicity (2000 mg/kg) assessed mortality, hematological, biochemical, histological parameters, water/feed intake, and body weight. Genotoxicity was evaluated via comet and micronucleus assays. Antitumor activity was tested in vitro and in vivo on sarcoma 180. SE contained 107.3 mg GAE/g phenolics and 22.9 mg QE/g flavonoids; the presence of gallic and ellagic acids was detected. Protein concentration was 12.16 mg/mL with lectin activity present. No mortality, organ damage, or genotoxic effects occurred in toxicity tests. SE demonstrated in vitro cytotoxicity against sarcoma cells (IC_50_: 10 µg/mL). In vivo, SE (50–200 mg/kg) reduced tumor weight by 70.2–72.3%. SE modulated IL-2, IL-4, IL-6, IL-17, IFN-γ, and TNF-α in tumor environment. Tumors showed inflammatory infiltrate, necrosis, and fibrosis after treatment. These findings position the extract as a promising candidate for further development as a safe, plant-based antitumor agent.

## 1. Introduction

Cancer is a broad term for many diseases and is a leading cause of death worldwide, accounting for about 10 million deaths in 2020, with approximately 20 million cases reported that year. Future projections by the World Health Organization (WHO) predict that the number of cases will rise to around 30 million within the next two decades [1]. Carcinogenesis is related to the lifelong accumulation of DNA mutations and an imbalance in the processes that repair them [2]. These mutations can be deleterious, neutral, or occasionally advantageous, driving the evolution of cancer cells [3]. Aberrant proliferation, survival, migration, and cell cycle control, along with significant metabolic diversity and plasticity, are some features that distinguish cancer from normal cells [4].

Current methods to control or eliminate cancer cells include surgery, chemotherapy, radiotherapy, and immunotherapy, which may be used alone or in combination. However, resistance to therapy and the development of metastatic disease remain major causes of morbidity and mortality across many cancer types [3]. Advances in cancer research have led to the development of numerous novel chemotherapeutic agents [5]. Drugs such as cyclophosphamide, doxorubicin, methotrexate, and paclitaxel are effective at killing cancer cells but, unfortunately, are also associated with toxicity to normal cells. This toxicity can cause permanent or long-lasting side effects in various organs, which may reduce treatment adherence and negatively impact patients’ quality of life [6].

Natural products with anticancer properties are promising candidates due to their expected low toxicity [7,8]. Among these, plants offer a vast arsenal of bioactive compounds, making phytotherapy an increasingly supported potential cancer treatment. Plant extracts rich in bioactive proteins and secondary metabolites have gained considerable attention for their role in cancer prevention and treatment [9,10,11]. Although plant extracts may not yet be regarded as treatments on par with chemotherapeutic agents, they hold significant value in complementary medicine, drug discovery, and potential therapeutic applications.

Plant products are often considered safe and low in toxicity based on their history of human use; however, this assumption can be misleading and potentially harmful. Therefore, to ensure their safe and effective use thorough toxicological research is necessary [12]. Acute toxicity tests can evaluate parameters such as weight loss or gain, behavioral changes, biochemical, hematological, and histopathological effects, as well as estimate the median lethal dose (LD_50_) [13,14]. Additionally, plant products may contain compounds that cause DNA damage and could be genotoxic; thus, a comprehensive toxicity assessment should include genotoxicity evaluation [15].

*Euphorbia hirta* L., also known as *Chamaesyce hirta* (L.) Millsp. (Euphorbiaceae), is a medicinal plant widely used globally. Its leaves, aerial parts, and whole plant are rich sources of phytochemicals. Traditional medicine practitioners commonly use aqueous extracts of various *E. hirta* parts orally for many purposes [16]. In India, this plant is used for tumor treatment [17,18]. Studies have demonstrated significant anticancer effects of *E. hirta* against various cancer cells, both through isolated compounds and solvent extracts [19]. In vitro anticancer activity has been reported for different *E. hirta* extracts against HepG2 (liver), MCF-7 (breast), and HL-60 (acute myeloid leukemia) cancer cell lines [20,21,22]. An aqueous extract of the whole plant showed anticancer activity in vivo using the Ehrlich Ascites Carcinoma model in mice [23].

Most literature focuses on aqueous extracts of *E. hirta*; however, using low salt concentrations during extraction can maximize the yield of primary metabolites, such as bioactive proteins like lectins, along with secondary metabolites (e.g., alkaloids, phenolic compounds, saponins, and glycosides). Therefore, saline extraction remains closely aligned with traditional usage while also enabling the recovery of both bioactive proteins and secondary metabolites. This study aimed to characterize the phytochemical composition and evaluate the acute toxicity and genotoxicity of the Saline Extract (SE) from *E. hirta* leaves in mice. Additionally, the potential of SE for cancer treatment was evaluated by assessing its in vivo effects in mice bearing solid subcutaneous sarcoma 180 tumors. Toxicological parameters were also measured following repeated-dose treatments with SE in these animals.

## 2. Results

Thin layer chromatography demonstrated the presence of phenolic compounds, terpenes/steroids, coumarins, and saponins in SE. The extract exhibited a total phenolic content of 107.3 ± 0.7 mg GAE/g (gallic acid equivalents per gram) and a total flavonoid content of 22.9 ± 0.4 mg QE/g (quercetin equivalents per gram). It also showed a protein concentration of 12.16 mg/mL, and lectin activity was detected at 256 hemagglutinating activity units.

**Figure 1:**
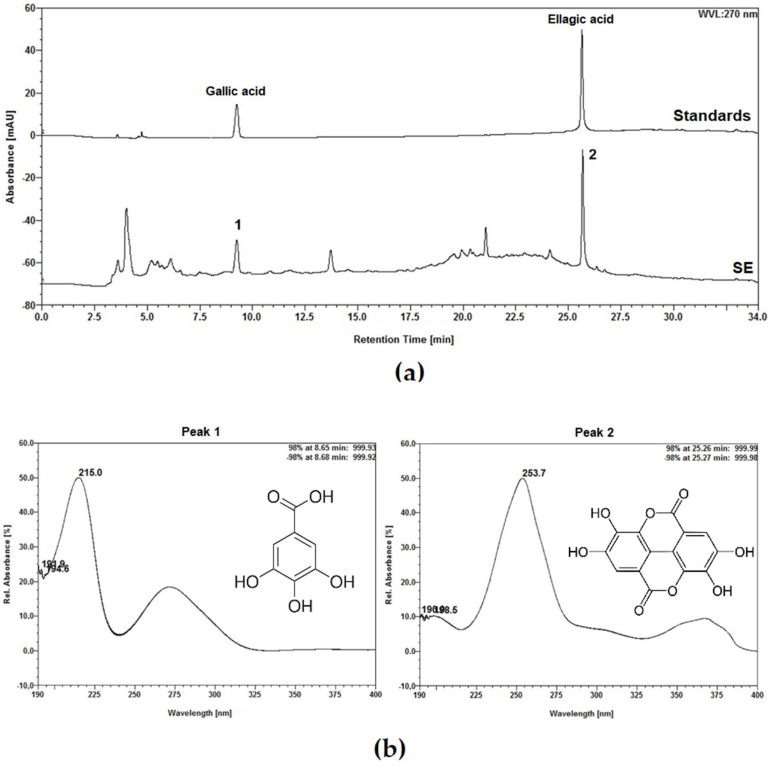
Phytochemical evaluation of Saline Extract (SE) from *Euphorbia hirta* leaves. (**a**) High-performance liquid chromatography (HPLC) profiles of SE and reference standards: gallic acid and ellagic acid. (**b**) UV spectra of the compounds detected in the SE. Peak 1 corresponds to gallic acid, and peak 2 corresponds to ellagic acid. HPLC profiles and UV spectra were recorded at 270 nm.

High-performance liquid chromatography (HPLC) analysis of SE at 270 nm produced a well-resolved chromatogram with distinct peaks, indicating the presence of multiple compounds, as shown in Figure 1. The chromatogram of standards is also shown in Figure 1. Gallic acid and ellagic acid were successfully identified at peaks 1 and 2, with retention times of 8.66 min and 25.27 min, respectively. Quantitative analysis determined the gallic acid content to be 0.239 ± 0.0021% (*w*/*v*) and the ellagic acid content to be 0.482 ± 0.0018% (*w*/*v*).

In the acute toxicity test, a single oral dose of SE (2000 mg/kg) caused no mortality or signs of intoxication. However, SE-treated animals showed a decrease in body weight gain compared to the control group, with no statistically significant differences in water or food consumption (Table 1).

None of the evaluated hematological parameters showed statistically significant differences between mice treated with a single dose of SE and the control group (Table 2). Regarding biochemical parameters, only the lipid profile showed significant differences. SE-treated mice exhibited reduced levels of triglycerides, low-density lipoprotein (LDL), and very-low-density lipoprotein (VLDL), while total cholesterol and high-density lipoprotein (HDL) levels showed no significant differences compared to control (Table 3).

**Figure 2:**
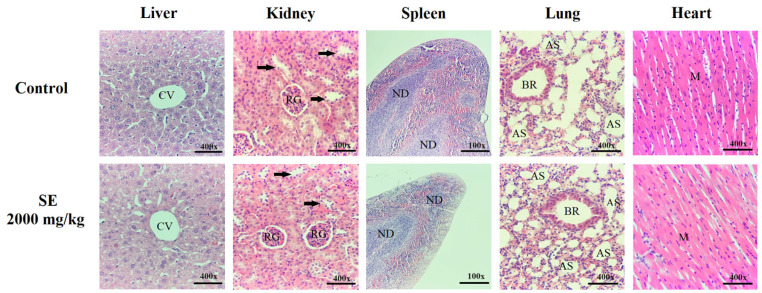
Histological sections of the liver, kidney, spleen, lung, and heart from control animals and those treated orally with a single dose (2000 mg/kg) of SE from *Euphorbia hirta* leaves. Liver: centrilobular vein (CV) visible in all images. Kidney: renal glomeruli (RG) and convoluted tubules (arrows) appear preserved and well-organized. Spleen: lymphatic nodules (ND) are well-defined in both groups. Lung: bronchioles (BR) and alveolar sacs (AS) maintain normal architecture. Heart: cardiac muscle (M) shows preserved structure throughout the myocardium. No signs of degeneration, necrosis, inflammation, or other pathological changes were observed. Hematoxylin and eosin staining; magnification: 400× for liver, kidney, lung, and heart; 100× for spleen.

Organ analysis showed that a single oral dose of SE did not cause significant changes in relative organ weights compared to the control group (Table 4). Additionally, no notable differences in macroscopic features, such as color or texture, were observed between the SE-treated and control groups. Microscopic examination revealed that both groups maintained normal, well-preserved organ architecture, with no harmful alterations or morphological disturbances induced by the treatment (Figure 2).

**Figure 3:**
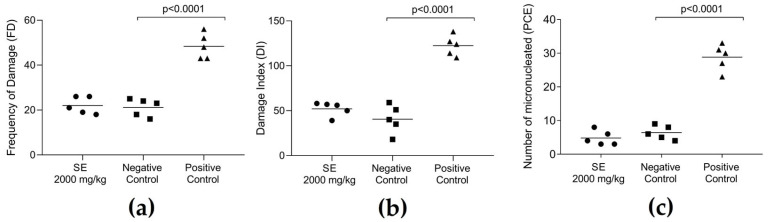
Evaluation of genotoxicity and mutagenicity in blood cells from animals of control groups (negative and positive) and those treated orally with a single dose (2000 mg/kg) of the SE from *Euphorbia hirta* leaves. Panels show frequency of damage (**a**), damage index (**b**), and number of micronucleated erythrocytes (**c**). Data are presented as mean ± SEM (*n* = 5 per group). Statistically significant differences (*p* < 0.05) are indicated on the bars compared to the positive control, using one-way ANOVA followed by Tukey’s test.

In addition to assessing acute oral toxicity, the genotoxicity of SE in vivo was evaluated to detect potential DNA damage or mutations in blood cells. Results from the comet assay (Figure 3) showed no significant differences between SE-treated mice and the negative control, with most leukocytes being classified as class 0 (no damage). Figure 3a shows that the frequency of damage was similar between the SE group (22.0 ± 1.70) and the negative control (21.20 ± 1.77), while the positive control showed significantly higher damage (48.40 ± 2.54). The damage index indicated that SE treatment (52.00 ± 3.53) was comparable to the negative control (40.60 ± 7.03) and much lower than the positive control (122.4 ± 5.08) (Figure 3b). The micronucleus test (Figure 3c) confirmed that SE was not mutagenic, as the number of micronucleated polychromatic erythrocytes in the SE group (4.80 ± 0.96) did not differ from the negative control (6.40 ± 0.92), unlike the positive control (methotrexate, MTX), which showed a significant increase (28.80 ± 1.74).

**Figure 4:**
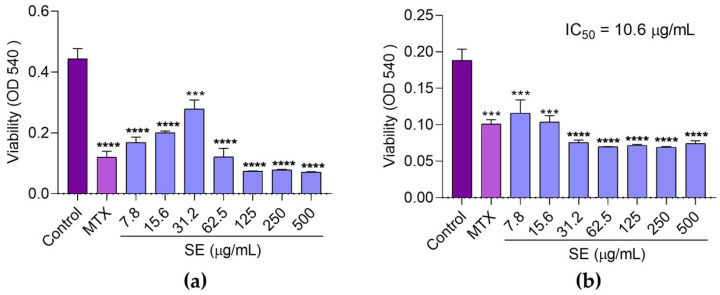
Cytotoxicity assessment of the SE from *Euphorbia hirta* leaves against sarcoma 180 cells using the MTT assay. Cells were treated with PBS (control), methotrexate (MTX, 20 µg/mL), or SE (7.8–500 µg/mL) and incubated for 48 h (**a**) and 72 h (**b**). Data are expressed as mean ± SEM. (***) *p* < 0.001 and (****) *p* < 0.0001 indicate significant differences compared to the control, as determined by one-way ANOVA followed by Tukey’s test. The IC_50_ value was calculated using non-linear regression.

In the antitumor evaluation, the in vitro cytotoxicity of SE against sarcoma 180 cells was first assessed. The results showed a significant reduction in cell viability compared to the control group, comparable to the effects of MTX (Figure 4). The IC_50_ value of SE was 10.6 µg/mL at 72 h.

**Figure 5:**
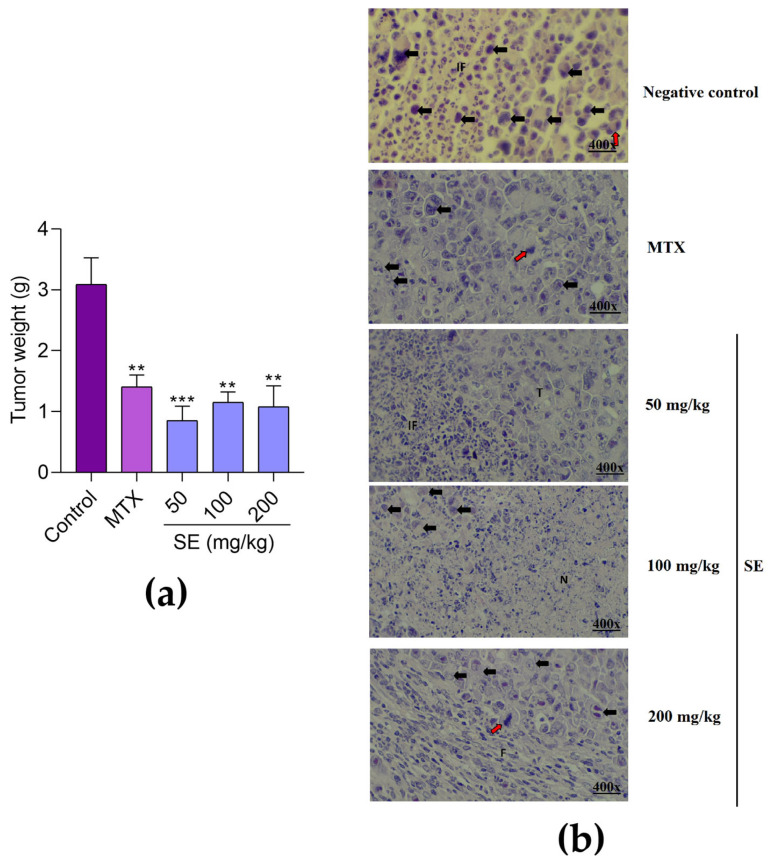
Evaluation of the in vivo antitumor activity of the SE from *Euphorbia hirta* leaves. (**a**) Tumor weight and (**b**) photomicrographs of sarcoma 180 tumors after 7 days of treatment with PBS (negative control, oral), methotrexate (MTX, positive control, 1.5 mg/kg, i.p.), or SE (50, 100, and 200 mg/kg, oral). Bars represent mean ± SEM (*n* = 6 per group). ** *p* < 0.01 and *** *p* < 0.001 indicate significant differences compared to the control, analyzed by one-way ANOVA followed by Tukey’s test. T: sarcoma 180 cells; black arrows: cells with varied sizes and nuclei; red arrow: mitotic figures; IF: inflammatory infiltrate; N: necrosis; F: fibrosis.

SE at doses of 50, 100, and 200 mg/kg caused significant tumor mass reductions of 72.32 ± 7.61%, 70.25 ± 8.47%, and 70.89 ± 9.76%, respectively, compared to the control (Figure 5a). The positive control, MTX, induced a significant tumor mass reduction of 54.46 ± 6.32% compared to the control.

Tumor photomicrographs of the experimental groups (Figure 5b) showed that the negative control exhibited sarcoma 180 cells with varied sizes and nuclei, mitotic figures, and diffuse inflammatory infiltrate. The MTX-treated group displayed sarcoma 180 cells with varied sizes, multinucleated cells, and mitotic figures. SE at 50 mg/kg showed sarcoma 180 cells with varied sizes and nuclei alongside inflammatory infiltrate. SE at 100 mg/kg revealed sarcoma 180 cells with large necrotic areas, while SE at 200 mg/kg presented sarcoma 180 cells with mitotic figures and regions of fibrosis.

**Figure 6:**
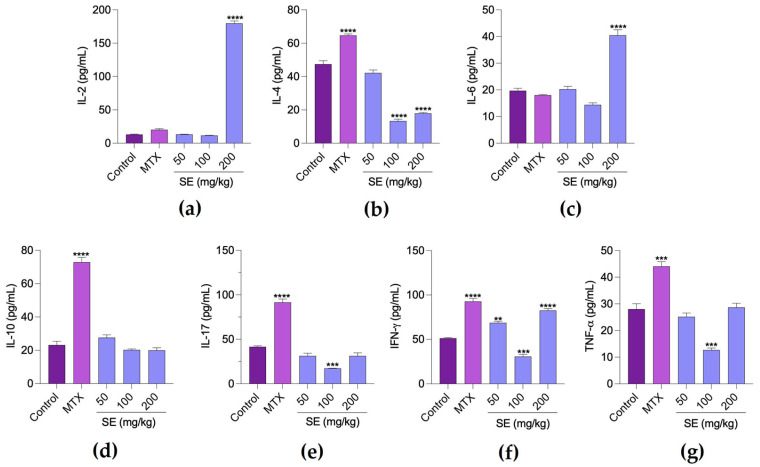
Levels of the cytokines interleukin (IL) 2 (**a**), IL-4 (**b**), IL-6 (**c**), IL-10 (**d**), IL-17 (**e**), interferon (IFN) γ (**f**), and tumor necrosis factor (TNF) α (**g**) in tumor homogenates from animals treated with PBS (negative control), methotrexate (MTX, positive control, 1.5 mg/kg, i.p.), and SE (50, 100, and 200 mg/kg, oral). Each bar represents the mean ± SEM of cytokine production from a tumor homogenate prepared per animal (*n* = 6 per group). ** *p* < 0.01, *** *p* < 0.001, and **** *p* < 0.0001 indicate significant differences compared to the control group, analyzed by one-way ANOVA followed by Tukey’s test.

Treatment with SE at 50 mg/kg did not significantly affect cytokine levels in the tumor microenvironment. However, SE at 100 mg/kg reduced IL-4, IL-17, IFN-γ, and TNF-α levels, indicating a predominantly anti-inflammatory profile. In contrast, SE at 200 mg/kg decreased IL-4 but increased IL-2, IL-6, and IFN-γ levels, suggesting a more pro-inflammatory response. MTX induced both anti-inflammatory (IL-4 and IL-10) and pro-inflammatory (IL-17, IFN-γ, and TNF-α) cytokines (Figure 6).

Repeated treatment of tumor-bearing animals with SE (100 and 200 mg/kg) for seven days reduced weight gain compared to the Sham group, with weight loss observed. However, water and food consumption were similar among all tumor-bearing and healthy mice (Table 5). This study found an increase in spleen weights of tumor-bearing animals (Table 6), along with a higher percentage of segmented cells and a reduced percentage of lymphocytes in their peripheral blood compared to healthy controls (Sham group).

Hematological analysis (Table 7) showed that MTX induced anemia in treated animals, evidenced by reductions in erythrocytes count, hematocrit, hemoglobin, and mean corpuscular hemoglobin concentration compared to the Sham group. Total leukocyte counts increased significantly only in the negative control and MTX-treated groups (Table 7). Biochemical parameters (Table 8) revealed that MTX increased liver enzymes—alanine aminotransferase, aspartate aminotransferase, and alkaline phosphatase—while decreasing albumin and total plasma protein levels relative to the sham group. Conversely, SE at doses of 50, 100, and 200 mg/kg did not cause any hematological or biochemical changes indicative of toxicity. However, as shown in Table 8, a hypolipidemic effect was observed, with reductions in total cholesterol and triglycerides at the highest doses compared to the Sham group.

**Figure 7:**
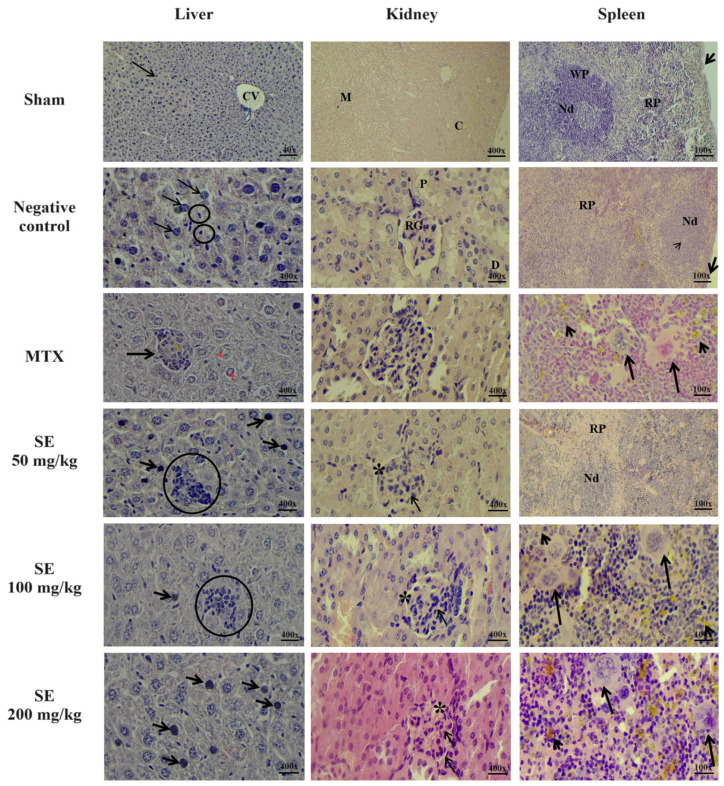
Histopathological analysis of the organs of healthy animals (Sham group) and sarcoma 180-bearing animals treated with PBS (negative control, *per os*), methotrexate (MTX, 1.5 mg/kg, i.p.), or SE (50, 100, and 200 mg/kg, *per os*). Liver photomicrographs show, in the Sham group, normal hepatocytes (arrow) and the centrilobular vein (CV); in the Negative Control group, multinucleated hepatocytes (arrows) and Kupffer cells (circle) are observed; the MTX group displays inflammatory infiltrate (arrow) and vacuolation (red asterisk); and the SE groups show inflammatory infiltrate (circle) and hepatocytes with pyknotic nuclei (arrow). Kidney photomicrographs include the cortical (C) and medullary (M) regions; renal glomerulus (RG); proximal (P) and distal (D) convoluted tubules; subcapsular space (black asterisk); and increased cellularity in the glomerulus (arrow). Spleen photomicrographs highlight the capsule (arrow), red pulp (RP), white pulp (WP), lymphoid nodules (Nd), central arteriole (thin arrow), megakaryocytes (long arrow), and macrophages (short arrow). All tissues were stained with hematoxylin and eosin.

Histopathological analysis of the liver from all experimental groups revealed a well-developed organ, covered by a thin mesothelial capsule and organized into lobules containing veins of varying sizes. The parenchyma consisted of hepatocytes arranged around the centrilobular vein, characterized by polyhedral morphology, central nuclei, prominent nucleoli, and acidophilic cytoplasm. Sinusoidal capillaries with Kupffer cells were also observed among the hepatocytes. However, the MTX group showed a small inflammatory infiltrate and early signs of vacuolation, and the SE-treated groups (50, 100, and 200 mg/kg) exhibited a small inflammatory infiltrate and hepatocytes with pyknotic nuclei (Figure 7).

Morphological analysis of the kidneys from all experimental groups showed an intact capsule covering the organ and well-defined cortical and medullary regions. In the cortex, renal glomeruli and proximal and distal convoluted tubules were observed. However, the MTX- and SE-treated groups (50, 100, and 200 mg/kg) exhibited a reduced subcapsular space, narrowed tubular lumens, and increased cellularity in the glomeruli compared to the other groups (Figure 7).

Histological analysis of the spleen revealed an external capsule and well-defined red and white pulp regions, with the white pulp consisting of lymphoid nodules containing germinal centers and central arterioles. Megakaryocytes were present in the red pulp. The SE-treated groups showed disorganization of the lymphoid nodules, increased numbers of megakaryocytes and leukocytes in the red pulp, and vacuolation compared to the other groups (Figure 7).

## 3. Discussion

This study demonstrates that saline extraction from *E. hirta* leaves could effectively capture lectins and different classes of secondary metabolites (including gallic and ellagic acids). In this context, SE was assessed for acute toxicity, genotoxicity, and antitumor potential in vivo. The toxicological outcomes observed further contribute to understanding the safety profile of SE in a preclinical setting.

Extensive research on different species of *Euphorbia* has revealed a broad spectrum of biologically active phytochemical constituents, including tannins, flavonoids, alkaloids, phenolic compounds, coumarins, cyanogenic glycosides, sterols, triterpenes, and diterpenes [24,25]. Among these, phenolic compounds have emerged as significant markers of the phytochemical profile of *E. hirta* [23,26,27,28,29]. Consistent with these findings, our data also indicate that *E. hirta* leaves are rich in phenolic and flavonoid compounds. In a study by Mekam et al. [26], aqueous and ethanolic leaf extracts of *E. hirta* revealed the presence of 123 individual phenolic compounds and 18 non-phenolic phytochemicals. The aqueous extract showed a notably higher concentration of phenolics (163.62 ± 0.61 mg/g) compared to the ethanolic extract (49.61 ± 0.39 mg/g). Das et al. [29] reported that the ethanolic leaf extract of *E. hirta* contained a total phenol content of 143.20 ± 0.21 mg GAE/g and a total flavonoid content of 87.53 ± 0.30 mg QE/g, both values higher than those determined here for SE.

Gallic acid and ellagic acid were identified in SE and these compounds, along with their derivatives, have also been reported as phytoconstituents in the aqueous extract of the whole *E. hirta* plant [23] and in both aqueous and ethanolic leaf extracts of *E. hirta* [26]. Gallic acid has garnered significant attention in the pharmaceutical and chemical industries due to its wide range of biological activities, including antioxidant, anticancer, antibacterial, antifungal, antiviral, anti-inflammatory, and antidiabetic properties [29,30,31]. Ellagic acid, a polyphenolic compound and dimeric derivative of gallic acid, also exhibits strong antioxidant activity, anti-adipogenic effects, and shows considerable potential in cancer prevention and treatment [32,33,34,35]. Both compounds have attracted interest for their diverse therapeutic applications across various biomedical fields.

Additionally, it was detected in SE the presence of lectins, which are proteins that can recognize and reversibly bind to glycoconjugates [10]. Lectins have been widely recognized for their diverse pharmacological activities, including neuromodulatory [36], anti-inflammatory [37], antimicrobial [38], and antitumor [39] effects, for example. The presence of lectins in the extract not only supports the effectiveness of the saline extraction method in preserving functional proteins but also highlights the potential of *E. hirta* as a source of protein-based bioactives. Their detection opens promising avenues for further studies into the molecular mechanisms through which these proteins may contribute to the observed biological effects.

SE also contains other pharmacologically relevant constituents that were not fully identified using the analytical methods applied in this study. In addition, compounds present in low concentrations, or those with complex or unstable chemical structures may have gone unnoticed. Identifying and characterizing these components would require complementary analytical approaches, including liquid chromatography–mass spectrometry, nuclear magnetic resonance spectroscopy, or targeted proteomic techniques. Future investigations employing these advanced methodologies could significantly expand our understanding of the full phytochemical profile of SE.

Once concluded the phytochemical analysis, the extract was initially assessed for acute toxicity, during which signs of toxic effects were not observed. Similarly, the aqueous extract of the whole *E. hirta* plant, when administered at the same dose (2000 mg/kg) in Wistar rats, showed no mortality or alterations in body weight or clinical behavior [23]. These findings are in line with a study involving the methanolic extract of *E. hirta* leaves at 3200 mg/kg, which reported no signs of toxicity, behavioral changes, or mortality [40]. In contrast, administration of the methanolic extract at a higher dose (5000 mg/kg) led to one-third mortality and mild behavioral changes in mice [41].

Mice treated with SE exhibited a hypolipidemic effect, consistent with previous findings in Wistar albino rats treated with various doses (200, 400, and 600 mg/kg) of ethanolic *E. hirta* leaf extract for 14 days, which resulted in a significant reduction in serum lipid parameters, including triglycerides, LDL, VLDL, and total cholesterol, compared to control [42]. Gallic acid has demonstrated notable anti-adipogenic properties and cardioprotective activity [43,44,45]. In a study where gallic acid (100 mg/kg) was orally administered to obese Swiss mice, significant reductions in body weight and adipose tissue mass were observed compared to animals fed a high-fat diet. Additionally, the treatment led to decreased total cholesterol levels and improved glucose tolerance [46]. The anti-obesity effects of gallic acid are attributed to its ability to inhibit leptin secretion, reduce triglycerides, LDL, and VLDL levels, while simultaneously increasing HDL levels [47]. Furthermore, ellagic acid, which is also present in SE, has been reported to attenuate the formation of new adipocytes and inhibit fatty acid biosynthesis in adipose tissue. It also reduces triglyceride and fatty acid synthesis while enhancing fatty acid oxidation in the liver [48].

Similarly to the data found for SE, the methanolic leaf extract [41] and the methanolic extract of the whole *E. hirta* plant [49] did not affect organ weight or histopathological features in acute toxicity assays. The results of the acute toxicity study indicated a LD_50_ > 2000 mg/kg for SE, which was then considered non-toxic according to the Globally Harmonized System of Classification and Labelling of Chemicals (GHS). Supporting this classification, previous studies have reported LD_50_ values greater than 5000 mg/kg for methanolic extracts of the whole plant [49] and leaves [41] of *E. hirta*.

However, there is a lack of literature reporting the evaluation of the genotoxic effects of *E. hirta* extracts using in vivo models, and the present study is among the first to provide data on the genotoxicity of this species. Together, the results from comet and micronucleus assay support the genetic safety of the SE at the tested dose. The absence of genotoxic and mutagenic effects is particularly relevant in the context of developing plant-based therapies, as it provides a critical indication of the extract’s safety profile for potential therapeutic use.

Since no evidence of toxicity of SE in mice was found, the investigation proceeded to evaluate its antitumor potential, beginning with an assessment of in vitro cytotoxicity against sarcoma 180 cells. SE proved to be cytotoxic to cancer cells, similarly to other extracts and compounds from *E. hirta.* The ethanolic extract of *E. hirta* leaves was evaluated against Dalton Lymphoma Ascites (DLA) and Ehrlich Ascites Carcinoma (EAC) cell lines yielding IC_50_ values of 560.83 μg/mL for DLA and 384.7 μg/mL for EAC at 24 h [50]. The methanolic extract of the whole *E. hirta* plant showed significant inhibition of MCF-7 breast cancer cell survival, with an IC_50_ value of 25.26 μg/mL at 24 h [21]. Additionally, flavonol glycosides afzelin, quercetrin, and myricitrin—isolated from the methanolic extract of *E. hirta* aerial parts—demonstrated cytotoxic effects against human epidermoid carcinoma KB 3–1 cells, with IC_50_ values of 276.1, 88.2, and 156.4 μg/mL, respectively [51]. Two terpenoids isolated from the ethanolic extract of *E. hirta* leaves, 5-hydroperoxycycloart-23-en-3β-ol and 4-hydroperoxycycloart-25-en-3β-ol, exhibited cytotoxicity against human cancer cell lines, with IC_50_ values of 4.8 μg/mL for colon carcinoma (HCT 116) and 4.5 μg/mL for non-small cell lung adenocarcinoma (A549) [52]. Moreover, the whole *E. hirta* ethanolic extract showed significant cytotoxicity against acute myeloid leukemia (HL-60) cells, with an IC_50_ of 100 μg/mL [20].

The in vitro cytotoxicity of gallic acid has been extensively studied across a variety of cancer cell lines. Its primary anti-tumor mechanisms involve inhibiting cell proliferation and inducing apoptosis while exhibiting minimal effects on normal cells [53]. Ellagic acid is similarly recognized for its potent anticancer properties. Research has shown that ellagic acid induces apoptosis in HT-29 colon cancer cells and human breast cancer MCF-7 cells [54,55]. Lectins have also been reported as active agents of extracts with anticancer activity. A saline leaf extract of *Schinus terebinthifolia* containing lectin exhibited an IC_50_ value of 301.65 μg/mL against sarcoma 180 cells, while the isolated lectin (SteLL) showed a much stronger cytotoxic effect with an IC_50_ of 8.30 μg/mL and was capable of inducing apoptosis [10].

The antitumor potential of SE indicated by in vitro assay was confirmed by in vivo assays using sarcoma 180. So far, Sulaiman et al. [23] have evaluated the anticancer activity of *E. hirta* in vivo, focusing on Ehrlich ascites carcinoma induced as peritoneal ascites in mice, rather than using a solid tumor model. Research has shown that ellagic acid reduces the incidence of lateral prostatic adenocarcinoma and suppresses the progression of prostate carcinogenesis [56], and lectins have been reported to disrupt the development of sarcoma 180 in mice [10,39].

The findings from this study highlight the potential therapeutic value of SE in tumor-bearing animals, particularly due to its apparent safety profile and biological activity. Unlike methotrexate (MTX), SE did not induce hematological or biochemical toxicity, suggesting a more favorable safety margin. This is especially relevant in the context of cancer therapy, where treatment-associated toxicity often limits clinical outcomes. While mild hepatic and renal histological changes were observed at higher SE doses, these did not coincide with corresponding biochemical markers of organ dysfunction, suggesting subclinical or early-stage alterations. Future studies should assess the reversibility of these changes and determine whether they progress with prolonged treatment.

SE’s impact on lipid metabolism was also observed in tumor-bearing animals, raising additional interest in its potential as a metabolic modulator. This effect may contribute to systemic improvements in the tumor-bearing host or reflect underlying anti-inflammatory mechanisms. Interestingly, the reduction in weight gain observed with SE treatment, both in the acute toxicity assay and in tumor-bearing mice, suggests potential metabolic effects that warrant further investigation.

Histological changes in lymphoid organs, particularly the spleen, suggest that SE may influence immune function. Given the central role of the immune system in tumor surveillance and response to therapy, this potential immunomodulatory effect was investigated by assessing cytokine levels in the tumor microenvironment. Tumors escape immune surveillance by developing an immunosuppressive microenvironment that induces immune tolerance and tumor progression. Meanwhile, inflammation in the tumor microenvironment is an incubator for cancer progression and therapy resistance. Immunosuppressive cytokines such as IL-4 and IL-10 promote differentiation of monocytes to tumor-associated macrophages and regulatory T cells, thereby inhibiting T-cell effector functions and contributing to a protumorigenic environment. In contrast, Th1 cytokines, including IL-2 and IFN-γ, favor activation of cytotoxic T lymphocytes and NK cells, supporting an anti-tumor immune response [57]. In this context, our results suggest that lower doses of SE may maintain an immunosuppressive tumor microenvironment, while higher doses shift the balance toward a pro-inflammatory, tumor-suppressive profile. The immunomodulatory effects observed may significantly contribute to the extract’s antitumor activity. Similarly, immune-mediated antitumor mechanisms have been described for other plant extracts, such as *Panax ginseng* fruit [58] and *Ventilago leiocarpa* Benth. [59].

Overall, SE demonstrated promising biological activity without the myelosuppressive and hepatotoxic side effects commonly associated with MTX. These findings support further investigation of SE as a potential adjuvant or alternative therapeutic strategy in cancer, with particular attention to its immunological and metabolic effects.

## 4. Materials and Methods

### 4.1. Plant Material and Extract Preparation

Leaves of *E. hirta* (species name verified via http://www.worldfloraonline.org; accessed on 16 June 2025) were collected at the *Centro Acadêmico do Agreste*, *Universidade Federal de Pernambuco* (UFPE), Caruaru, Pernambuco, Brazil (8°22′58.5″ S, 35°98′14.6″ W). A voucher specimen (no. 88608) was deposited in the Herbarium Dárdano de Andrade Lima at the *Instituto Agronômico de Pernambuco* in Recife, Brazil. This research was also registered under code AF214DD in the *Sistema Nacional de Gestão do Patrimônio Genético e do Conhecimento Tradicional Associado* (SisGen).

For extract preparation, the leaves were thoroughly washed with running water and air-dried at 25 °C for 7 days. The dried leaves were then ground into a fine powder, which was homogenized (10 g) in saline solution (0.15 M NaCl) buffered with 100 mM sodium phos phate pH 7.5 (100 mL). Extraction was carried out for 4 h at 28 °C under magnetic stirring (Thermo Fisher Scientific, Waltham, MA, USA). The mixture was filtered through a gauze and centrifuged (Thermo Fisher Scientific) at 9000× *g* for 15 min at 4 °C. The resulting supernatant constituted the saline extract (SE) from *E. hirta* leaves.

The SE was then extensively dialyzed against distilled water for 4 h and lyophilized using a LIOTOP L101 freeze-dryer (Liobras, São Carlos, Brazil) at −45 °C under a maximum vacuum of 300 μm Hg for 24 h. The dried extract was stored at −6 °C for further characterization and biological assays described in this study.

### 4.2. Phytochemical Characterization

#### 4.2.1. Thin Layer Chromatography

The lyophilized SE (1 g) was diluted in analytical grade methanol (20 mL). Standards were prepared and used at a concentration of 0.5 mg/mL. The samples and standards were manually applied to silica gel 60-F254 chromatographic plates (Macherey-Nagel, Düren, Germany). The plates were developed in chambers after saturation with the mobile phase [60]. The chamber was saturated for approximately 15 min at room temperature. After plate elution, they were dried at room temperature and observed under ultraviolet light at 254 and 365 nm, as well as under visible light. Then, they were revealed with specific reagents for each metabolite class: alkaloids, cinnamic derivatives, condensed tannins, coumarins, flavonoids, hydrolysable tannins, quinones, saponins, and sugars [60]. The bands obtained were compared to the corresponding standard bands.

#### 4.2.2. Total Phenol Content

The analysis of phenolic compounds was conducted following the method described by Li et al. [61], with minor modifications. An aliquot of SE (20 µL; 1 mg/mL) was combined with 100 µL of Folin–Ciocalteu reagent (10% *v*/*v*) in a 96-well plate. Subsequently, 80 µL of sodium carbonate solution (75 g/L) was added. The mixture was incubated in the dark at room temperature for 30 min. Absorbance was then measured at 765 nm using a spectrophotometer (Kasvi, Pinhais, Brazil).

#### 4.2.3. Total Flavonoid Content

The determination of flavonoid content was performed according to the method proposed by Woisky and Salatino [62], with slight modifications. In a 96-well plate, 100 µL of SE solution (1 mg/mL) was mixed with 100 µL of aluminum chloride reagent (20% *m*/*v*). The mixture was incubated in the dark for 1 h at room temperature, after which the absorbance was measured at 420 nm using a spectrophotometer. The total flavonoid content was quantified using a quercetin calibration curve (10–100 µg/mL) and expressed as milligrams of quercetin equivalents per gram of extract (mg QE/g extract).

#### 4.2.4. Analysis by High-Performance Liquid Chromatography (HPLC)

A solution was prepared by dissolving 20 mg of SE in 10 mL of methanol. From this stock solution, 5 mL aliquots were transferred into 10 mL volumetric flasks, followed by dilution with ultrapure water (Purelab Classic UV; ELGA LabWater, High Wycombe, UK). The diluted samples were then filtered into vials using 0.45 µm polyvinylidene difluoride (PVDF) syringe filters (Chromafil; Macherey-Nagel, Germany) prior to chromatographic analysis. HPLC analysis was carried out using an Ultimate 3000 system (Thermo Fisher Scientific) equipped with a diode array detector (DAD), binary pump (HPG-3 × 00RS), degasser, and autosampler (ACC-3000) with a 20 µL injection loop. The detection wavelength was set to 270 nm. Separation was achieved on a C18 analytical column (250 mm × 4.6 mm i.d., 5 µm; Supelco, Merck, Darmstadt, Germany) safeguarded by a matching C18 column (4 mm × 3.9 mm; Phenomenex, Torrance, CA, USA), maintained at 25 ± 1 °C. The mobile phase consisted of ultrapure water (solvent A) and methanol (solvent B), both containing 0.05% trifluoroacetic acid. A gradient elution was applied as follows:: 0–10 min, 5–20% B; 10–15 min, 20–25% B; 15–18 min, 25–40% B; 18–25 min, 40–80% B; 25–30 min, 80% B (isocratic); 30–34 min, 80–5% B. The flow rate was maintained at 0.8 mL/min throughout the run. Each sample was injected three times to ensure reproducibility. Data acquisition and processing were performed using Chromeleon 6.8 software (Dionex, Thermo Fisher Scientific). Quantification was based on calibration curves constructed with gallic acid (≥98%, Sigma-Aldrich, St. Louis, MO, USA) and ellagic acid (≥95%, Sigma-Aldrich) as reference standards.

#### 4.2.5. Protein Content and Hemagglutinating Activity Assay

The protein content of SE was determined following the method described by Lowry et al. [63], using bovine serum albumin (BSA) as a standard in the concentration range of 31.25–500 µg/mL. Hemagglutinating activity (HA) was evaluated using rabbit erythrocytes, according to the procedure outlined by Procópio et al. [64], to assess the presence of lectins. The hemagglutination titer was expressed in hemagglutinating units (HAU), calculated as the reciprocal of the highest sample dilution that produced complete erythrocyte agglutination.

### 4.3. Animals

The experiments were conducted using 61 female Swiss albino mice (*Mus musculus*) sourced from the animal facility of *Instituto Keizo Asami* (iLIKA) of UFPE. Sample sizes was decided based on The Organization for Economic Co-operation and Development (OECD) guidelines or commonly adopted experimental designs in similar in vivo studies evaluating genotoxicity and antitumor effects, considering the feasibility for statistical analysis. Inclusion criteria were animals aged 6 to 8 weeks and weighing 30–35 g. Animals were housed under controlled conditions at 22 ± 2 °C, with a 12 h light/dark cycle and relative humidity maintained between 50 and 60%. Food and water were provided ad libitum until the start of the experiments, after which each cage was supplied with 100 g of food and 200 mL of water to monitor individual consumption. All experimental protocols received approval from the Ethics Committee on Animal Use at UFPE (protocol numbers 0026/2022 and 0009/2023). All animals were included in the final analysis, with no need for any exclusions. To prevent confounders, all treatments were carried out in the following sequence: control and treated groups, in increasing order of dose. Only the researcher responsible for the administration was aware of the group allocation at the different stages. The collection of rabbit erythrocytes was also authorized under process number 23076.033782/2015-70.

### 4.4. Acute Toxicity Assessment

Acute toxicity was assessed following the OECD guideline No. 423 [65]. A total of ten female Swiss albino mice were randomly divided into two groups (*n* = 5 per group) using a simple randomization method. One group received a single oral dose of SE (dissolved in phosphate-buffered saline, PBS) at 2000 mg/kg body weight while the control group was administered PBS. Both treatments were delivered via oral gavage using a 14-gauge feeding needle. Animals were closely monitored for behavioral and clinical signs of toxicity continuously for the initial 2 h post-dosing, followed by daily observations for a period of 14 days. On the 14th day, animals were anesthetized with an intraperitoneal injection of ketamine (100 mg/kg) and xylazine (10 mg/kg) for blood collection via cardiac puncture for hematological and biochemical evaluations. Euthanasia was performed using the same anesthetic combination. Subsequently, major organs—including the liver, kidneys, spleen, lungs, and heart—were collected for gross pathological assessment and histological examination.

#### 4.4.1. Evaluation of Animal Weight, Water and Food Consumption, and Organ Weight

Animal body weight, organ weight, and food consumption were measured daily using a semi-analytical balance, while water intake was recorded with a graduated cylinder. Next, the following parameters were calculated using Equations (1)–(4):

Relative food consumption (%) = (Food consumed [g]/Body weight [g]) × 100(1)

Relative water consumption (%) = (Water consumed [mL]/Body weight [g]) × 100(2)

Relative organ weight (%) = (Organ weight [g]/Body weight [g]) × 100(3)

Body weight gain (g) = (Final body wight [g]/Initial body weight [g])(4)

#### 4.4.2. Evaluation of Hematological and Biochemical Parameters

For biochemical analysis, blood was drawn into tubes containing a separation gel and centrifuged at 450× *g* for 10 min to isolate the serum. The serum was then used to measure biochemical markers including albumin, alanine aminotransferase (ALT), aspartate aminotransferase (AST), alkaline phosphatase (ALP), total and direct bilirubin, gamma-glutamyl transferase (GGT), total protein, blood urea, creatinine, total cholesterol, triglycerides, high-density lipoprotein (HDL), low-density lipoprotein (LDL), and very low-density lipoprotein (VLDL). These parameters were quantified using commercial assay kits from Labtest Diagnóstica (Lagoa Santa, Brazil) on a COBAS Mira Plus automated analyzer (Hoffmann–La Roche, Basel, Switzerland). For hematological evaluation, blood samples were collected in EDTA-containing tubes and analyzed using an automated hematology analyzer (ABC Vet, Montpellier, France). The assessed parameters included erythrocyte count, leukocyte count, hemoglobin concentration, hematocrit, mean corpuscular volume (MCV), mean corpuscular hemoglobin (MCH), mean corpuscular hemoglobin concentration (MCHC), platelet count, as well as differential leukocyte counts such as segmented neutrophils, lymphocytes, monocytes, basophils, and eosinophils. Differential white cell counts were confirmed manually using light microscopy (Eclipse E100, Nikon, Tokyo, Japan).

#### 4.4.3. Histopathological Analysis

Histological analyses were conducted on liver, kidney, spleen, lung, and heart tissues from all experimental groups. Organ samples were fixed in 10% buffered formalin for 24 h, followed by dehydration in ethanol, clearing in xylene, and paraffin embedding. Sections of 3 µm thickness were cut using a Leica RM 25 RT microtome (Leica Biosystems, Nußloch, Germany) and stained with hematoxylin and eosin (Sigma-Aldrich, USA). Microscopic examination was performed with a light microscope (Eclipse E100, Nikon, Tokyo, Japan), capturing images at 400× magnification for the liver, kidney, lung, and heart, and at 100× magnification for the spleen.

### 4.5. Genotoxicity Assessment

A total of 15 mice (*n* = 5 per group) were randomly divided (simple randomization) into three experimental groups. The treatment group received SE (dissolved in PBS) orally at a dose of 2000 mg/kg, selected based on the absence of evidence of acute toxicity in prior tests. The negative control group was administered PBS orally, while the positive control group received methotrexate (MTX) intraperitoneally at 20 mg/kg.

#### 4.5.1. Comet Assay

The comet assay was conducted according to the method described by Singh et al. [66], with slight modifications. At 24 h post-treatment, 60 µL of whole blood was collected from the tail vein of each mouse. A 20 µL aliquot was combined with 110 µL of 0.5% low-melting-point agarose at 37 °C and immediately spread onto microscope slides pre-coated with a layer of normal-melting agarose. Coverslips were placed over the gels, which were allowed to solidify at 4 °C for 30 min. Subsequently, the coverslips were removed, and the slides were submerged in a cold lysis solution (2.5 M NaCl, 100 mM EDTA, 10 mM Tris, pH 10.0) for 1 h at 4 °C. Electrophoresis was then performed under alkaline conditions at 4 °C for 20 min at 32 V and 300 mA. After electrophoresis, the slides were neutralized in 0.4 M Tris buffer (pH 7.5) for 15 min, followed by dehydration in absolute ethanol for 5 min. The nucleoids were stained with 50 µL of propidium iodide (20 µg/mL) and visualized using a fluorescence microscope (Zeiss-Imager M2; Carl Zeiss AG, Jena, Germany). For each treatment group, 100 nucleoids were analyzed. Cells were categorized into five classes (0–4) based on DNA migration patterns, with class 0 indicating no damage and class 4 representing maximal damage. A damage index (DI) was calculated by multiplying the number of nucleoids in each class by the corresponding class value and summing the results. The frequency of DNA damage (FD%) was expressed as the percentage of cells displaying tail formation (classes 1–4) relative to the total number of cells scored. All experiments were independently replicated three times.

#### 4.5.2. Micronucleus Test

The micronucleus test was conducted 48 h post-treatment. Whole blood (60 µL) was collected from the tail vein of each mouse by venipuncture. A 5 µL aliquot of the collected blood was placed on slides previously stained with 8 µL of acridine orange (1 mg/mL) and covered with a coverslip [67]. Micronuclei were evaluated in 2000 polychromatic erythrocytes per animal using a Zeiss-Imager M2 fluorescence microscope equipped with a 40× objective and an Alexa Fluor 488 filter.

### 4.6. Evaluation of Antitumor Activity

#### 4.6.1. In Vitro Evaluation of Cytotoxicity to Sarcoma 180 Cells by MTT Assay

Murine sarcoma 180 (S-180) cells (1 × 10^7^ cells/mL, 0.3 mL) were inoculated intraperitoneally in mice, resulting in the development of ascites. Abdominal swelling was observed after approximately one week, indicating tumor cell proliferation. The cytotoxicity assay followed the protocol described by Ramos et al. [10]. Two milliliters of tumor ascitic fluid were collected via syringe from the peritoneal cavity, washed with PBS, and centrifuged at 150× *g* for 3 min at 22 °C. The supernatant was discarded, and the cell pellet was resuspended in RPMI 1640 medium with HEPES (Cultilab, Campinas, Brazil), supplemented with 10% fetal bovine serum (FBS; Sigma-Aldrich). Cell viability was assessed using the trypan blue exclusion test [68] with a Neubauer chamber. Cells were then plated at 1 × 10^5^ cells/mL per well in 96-well microplates. Serial dilutions of SE (7.8 to 500 µg/mL) were applied, with MTX (20 µg/mL) as the positive control and medium alone as the negative control. Plates were shaken to homogenize and incubated at 37 °C in a 5% CO_2_ atmosphere for 48 and 72 h. After incubation, plates were centrifuged at 2500 rpm for 5 min, supernatants removed, and 100 µL of Thiazolyl Blue Tetrazolium Bromide (MTT) solution (5 mg/mL in PBS) was added to each well. The plates were incubated for 3 h at 37 °C. Formazan crystals were solubilized by adding 100 µL of PBS mixed with 100 µL of DMSO, followed by shaking at 50 rpm for 10 min at 37 °C. Absorbance was measured at 540 nm using a microplate spectrophotometer. The IC_50_, representing the concentration that reduces cell viability by 50%, was determined by non-linear regression analysis using GraphPad Prism 9 software (GraphPad Software, Inc., San Diego, CA, USA).

#### 4.6.2. In Vivo Antitumor Evaluation

Thirty-six mice were randomly assigned (simple randomization) to six groups of six animals each. Sarcoma 180 cells (5 × 10^6^ cells/100 µL) suspended in PBS were subcutaneously inoculated into the dorsal region of all mice, except those in the sham group. Treatments commenced on day 8 post inoculation and continued daily for 7 days. The Sham and negative control groups received PBS orally, while the positive control group was administered MTX intraperitoneally at 1.5 mg/kg. The remaining three groups received oral doses of SE at 50, 100, or 200 mg/kg (dissolved in PBS). Animal body weight, as well as water and food consumption, were monitored daily as previously described (Section 4.4.1). On day 15 post inoculation, animals were euthanized via intraperitoneal injection of ketamine (100 mg/kg) and xylazine (10 mg/kg) (Syntec, Santana de Parnaíba, Brazil). Blood samples were collected by cardiac puncture for hematological and biochemical analyses as detailed in Section 4.4.2. Subsequently, tumor tissue along with lungs, liver, kidneys, spleen, heart, and stomach were excised, weighed, and processed for histopathological evaluation according to the protocol described in Section 4.4.3.

#### 4.6.3. Evaluation of Cytokine Levels in Tumor Microenvironment

Following tumor excision, 0.2 g of each tumor was sectioned and homogenized in 1 mL of PBS containing a protease inhibitor cocktail (Sigma-Aldrich) using vortex agitation until complete dissolution was achieved. Cytokine levels in tissue homogenates were measured using the Cytometric Bead Array (CBA) Mouse Th1/Th2/Th17 Cytokine Kit II (BD Biosciences, USA), following the manufacturer’s instructions. This multiplex assay enabled the simultaneous quantification of interferon-gamma (IFN-γ), interleukins IL-2, IL-4, IL-6, IL-10, IL-17, and tumor necrosis factor-alpha (TNF-α). Samples were analyzed on a BD Accuri C6 flow cytometer (BD Biosciences, Franklin Lakes, NJ, USA). Standard curves for each cytokine were prepared over a concentration range of 0–5000 pg/mL. Data were acquired and interpreted using BD Accuri C6 analysis software version 227.4. Samples for each animal were read three times in the cytometer.

### 4.7. Statistical Analysis

Results are expressed as the mean ± standard error of the mean (SEM) of replicates. Data normality was evaluated using the Shapiro–Wilk test. Acute toxicity assay results were analyzed by Student’s *t*-test, while antitumor assay data were assessed by one-way analysis of variance (ANOVA) followed by Tukey’s post hoc test. All statistical analyses were performed using GraphPad Prism version 9 (GraphPad Software, San Diego, CA, USA). Differences were considered statistically significant at *p* ≤ 0.05.

## 5. Conclusions

This study provides compelling preclinical evidence that SE from *E. hirta* leaves is both safe and biologically active, with significant antitumor potential. Rich in phenolics, flavonoids, and bioactive proteins, SE demonstrated no signs of toxicity or genotoxicity in vivo, even at high doses. Most notably, SE induced potent cytotoxicity against sarcoma 180 cells and achieved over 70% tumor weight reduction in mice, accompanied by immunomodulatory effects and histopathological changes indicative of tumor regression. These findings position its extract as a promising candidate for further development as a safe, plant-based antitumor agent. Future studies are warranted to elucidate in more detail its chemical composition and molecular mechanisms and to evaluate its efficacy in clinical models.

## Figures and Tables

**Table 1:** Relative consumption of food and water, and body weight gain of animals from control group or treated orally with a single dose (2000 mg/kg) of Saline Extract (SE) from *Euphorbia hirta* leaves.

Parameter	Treatment	
	Control	SE (2000 mg/kg)
Body weight gain (g)	2.40 ± 0.24	0.50 ± 0.87 *
Food consumption (%)	15.26 ± 0.83	14.00 ± 0.57
Water consumption (mL × 100)	23.00 ± 0.39	22.00 ± 0.87

Each value corresponds to the mean ± SEM of data from five animals (*n* = 5 per group). (*) *p* < 0.05 indicates significant differences between the groups evaluated by *t* test.

**Table 2:** Hematological parameters of animals from the control group or treated orally with a single dose (2000 mg/kg) of SE from *Euphorbia hirta* leaves.

Parameter	Treatment	
	Control	SE (2000 mg/kg)
Erythrocytes (10^6^/mm^3^)	5.77 ± 0.49	5.41 ± 0.53
Hematocrit (%)	38.76 ± 2.56	39.24 ± 3.03
Hemoglobin (g/dL)	14.89 ± 0.33	14.20 ± 0.27
Mean Corpuscular Volume (%)	48.11 ± 3.49	47.11 ± 3.71
Mean Corpuscular Hemoglobin (%)	17.39 ± 0.27	18.06 ± 0.32
Mean Corpuscular Hemoglobin Concentration (%)	37.18 ± 2.87	36.83 ± 3.04
Platelets (10^3^/mm^3^)	907.66 ± 77.19	926.70 ± 81.92
Leukocytes (10^3^/mm^3^)	6.23 ± 0.64	6.39 ± 0.40
Segmented Leukocytes (%)	70.70 ± 4.32	71.14 ± 4.57
Lymphocytes (%)	25.89 ± 0.30	25.13 ± 0.38
Monocytes (%)	3.10 ± 0.32	3.15 ± 0.36
Basophil (%)	0.10 ± 0.03	0.12 ± 0.03
Eosinophil (%)	1.56 ± 0.18	1.50 ± 0.14

Values represent the mean ± SEM (*n* = 5 per group). There were no significant differences between the groups evaluated by *t* test.

**Table 3:** Blood biochemical parameters of animals from the control group or treated orally with a single dose (2000 mg/kg) of SE from *Euphorbia hirta* leaves.

Parameter	Treatment	
	Control	SE (2000 mg/kg)
Albumin (g/dL)	35.10 ± 3.02	37.12 ± 3.33
Alanine aminotransferase (U/L)	69.77 ± 3.46	69.08 ± 4.15
Aspartate aminotransferase (U/L)	96.13 ± 4.47	94.69 ± 5.13
Alkaline phosphatase (U/L)	14.11 ± 0.64	14.34 ± 0.50
Bilirubin (mg/dL)	0.46 ± 0.12	0.48 ± 0.09
Gamma-glutamyl transferase (U/L)	13.56 ± 0.40	14.09 ± 0.54
Total protein (g/dL)	72.56 ± 4.10	73.89 ± 5.19
Blood urea (mg/dL)	0.38 ± 0.06	0.40 ± 0.09
Creatinine (mg/dL)	4.21 ± 0.54	4.09 ± 0.37
Total cholesterol (mg/dL)	73.30 ± 4.14	73.60 ± 4.55
Triglycerides (mg/dL)	97.11 ± 4.10	81.45 ± 4.12 *
High-density lipoprotein-cholesterol (mg/dL)	40.10 ± 2.10	41.13 ± 3.50
Low-density lipoprotein-cholesterol (mg/dL)	29.80 ± 2.01	21.09 ± 2.09 *
Very low density lipoprotein-cholesterol(mg/dL)	16.02 ± 1.16	12.60 ± 1.02 *

Values represent the mean ± SEM (*n* = 5 per group). (*) Significant differences (*p* < 0.05) were found between the groups evaluated by *t* test.

**Table 4:** Evaluation of the relative weight of organs of animals from the control group or treated orally with a single dose (2000 mg/kg) of SE from *Euphorbia hirta* leaves.

Relative Organ Weight (%)	Treatment	
	Control	SE (2000 mg/kg)
Heart	0.52 ± 0.03	0.44 ± 0.04
Kidney	1.38 ± 0.04	1.32 ± 0.07
Liver	5.79 ± 0.24	5.67 ± 0.29
Lung	0.85 ± 0.02	0.78 ± 0.05
Spleen	0.59 ± 0.03	0.57 ± 0.03

Values represent the mean ± SEM (*n* = 5 per group). No significant differences (*p* > 0.05) were found between the groups evaluated by *t* test.

**Table 5:** Mean of initial and final body weight and relative consumption of food and water of healthy animals (Sham group) as well as of sarcoma 180-bearing animals treated with PBS (negative control, *per os*), methotrexate (MTX, 1.5 mg/kg, i.p.) or SE (50, 100 and 200 mg/kg, *per os*).

Parameter	Groups					
	Sham	Negative Control	MTX	SE (50 mg/kg)	SE (100 mg/kg)	SE (200 mg/kg)
Body weight gain (g)	3.40 ± 0.48	4.00 ± 1.30	3.80 ± 0.66	1.20 ± 0.73	−0.83 ± 1.24 *	−0.66 ± 0.84 *
Food consumption (%)—day 1–7	18.64 ± 1.78	18.28 ± 0.77	19.27 ± 0.61	19.18 ± 0.54	17.88 ± 0.87	17.46 ± 0.87
Food consumption (%)—day 8–14	17.12 ± 1.17	16.46 ± 1.07	17.77 ± 0.84	14.89 ± 0.63	15.44 ± 1.19	14.34 ± 0.82
Water consumption (100 × mL)—day 1–7	27.70 ± 1.80	25.48 ± 0.85	29.37 ± 1.49	26.21 ± 1.86	25.5 ± 1.47	26.70 ± 1.71
Water consumption (100 × mL)—day 8–14	26.80 ± 2.10	23.94 ± 2.13	28.46 ± 2.28	22.10 ± 1.39	25.24 ± 2.54	22.52 ± 1.84

Values correspond to mean ± SEM (*n* = 6 per group). * *p* < 0.05 indicates significant differences in relation to the Sham group evaluated by one-way ANOVA followed by Tukey’s test.

**Table 6:** Evaluation of the relative weight of organs (%) of healthy animals (Sham group) as well as of sarcoma 180-bearing animals treated with PBS (negative control, *per os*), methotrexate (MTX, 1.5 mg/kg, i.p.), or SE (50, 100 and 200 mg/kg, *per os*).

Relative Organ Weight (%)	Treatment					
	Sham	Negative Control	MTX	SE (50 mg/kg)	SE (100 mg/kg)	SE (200 mg/kg)
Heart	0.47 ± 0.02	0.41 ± 0.02	0.42 ± 0.02	0.47 ± 0.02	0.43 ± 0.03	0.41 ± 0.01
Kidney	1.24 ± 0.04	1.28 ± 0.07	1.18 ± 0.05	1.28 ± 0.08	1.23 ± 0.03	1.09 ± 0.05
Liver	4.92 ± 0.26	5.70 ± 0.44	6.08 ± 0.45	5.08 ± 0.24	5.23 ± 0.39	4.84 ± 0.17
Lung	0.75 ± 0.01	0.70 ± 0.03	0.69 ± 0.04	0.76 ± 0.03	0.77 ± 0.08	0.77 ± 0.06
Spleen	0.43 ± 0.03	0.82 ± 0.08 *	1.00 ± 0.08 **	0.83 ± 0.09 *	0.85 ± 0.11 *	0.70 ± 0.07 *

Values correspond to mean ± SEM of data from six animals (*n* = 6/group). (*) *p* < 0.05 and (**) *p* < 0.01 indicates significant differences in relation to the Sham group evaluated by one-way ANOVA followed by Tukey’s test.

**Table 7:** Hematological parameters of healthy animals (Sham group) as well as of sarcoma 180-bearing animals treated with PBS (negative control, *per os*), methotrexate (MTX, 1.5 mg/kg, i.p.), or SE (50, 100 and 200 mg/kg, *per os*).

Parameter	Treatment					
	Sham	Negative Control	MTX	SE (50 mg/kg)	SE (100 mg/kg)	SE (200 mg/kg)
Erythrocytes (10^6^/mm^3^)	8.60 ± 0.15	7.60 ± 0.29	7.00 ± 0.33 **	8.60 ± 0.23	8.85 ± 0.25	8.41 ± 0.26
Hematocrit (%)	45.10 ± 0.75	39.83 ± 1.90	37.60 ± 1.74 **	45.33 ± 1.40	46.60 ± 1.35	43.33 ± 1.07
Hemoglobin (g/dL)	15.26 ± 0.20	13.32 ± 0.58	11.94 ± 0.62 ***	14.82 ± 0.44	15.06 ± 0.43	14.29 ± 0.37
Mean Corpuscular Volume (%)	52.81 ± 0.53	53.61 ± 1.40	54.89 ± 0.91	52.71 ± 0.83	52.67 ± 0.43	51.38 ± 0.51
Mean Corpuscular Hemoglobin (%)	17.75 ± 0.12	17.36 ± 0.47	17.39 ± 0.18	17.22 ± 0.13	17.02 ± 0.10	16.91 ± 0.22
Mean Corpuscular Hemoglobin Concentration (%)	33.63 ± 0.22	32.38 ± 0.25	31.17 ± 0.46 **	32.70 ± 0.43	32.32 ± 0.25	32.73 ± 0.28
Platelets (10^3^/mm^3^)	851.40 ± 80.0	735.33 ± 84.	818.20 ± 138.7	987.50 ± 230.2	970.00 ± 152.0	1160.00 ± 300.0
Leukocytes (10^3^/mm^3^)	5.07 ± 0.42	13.88 ± 2.24 *	21.58 ± 2.22 ****	11.12 ± 2.09	11.38 ± 2.18	9.84 ± 1.37
Segmented Leukocytes (%)	21.60 ± 2.10	61.00 ± 4.82 ***	66.80 ± 5.63 ****	49.33 ± 7.54 *	53.60 ± 8.18 **	66.43 ± 1.04 ***
Lymphocytes (%)	76.20 ± 2.27	36.33 ± 4.61 ***	31.60 ± 5.46 ***	46.33 ± 8.16 *	43.20 ± 8.51 **	27.87 ± 1.88 ****
Monocytes (%)	1.44 ± 0.67	1.60 ± 0.24	1.92 ± 0.71	2.40 ± 0.51	2.92 ± 0.71	3.55 ± 0.80
Basophil (%)	0.60 ± 0.89	0.00 ± 0.00	0.00 ± 0.00	0.55 ± 0.32	0.48 ± 0.12	0.22 ± 0.16
Eosinophil (%)	0.33 ± 0.21	1.00 ± 0.42	0.66 ± 0.81	0.66 ± 0.33	0.60 ± 0.32	0.16 ± 0.17

Values represent the mean ± SEM (*n* = 6 per group). (*) *p* < 0.05, (**) *p* < 0.01, (***) *p* < 0.001, and (****) *p* < 0.0001 indicate significant differences between the groups evaluated by *t* test.

**Table 8:** Blood biochemical parameters of healthy animals (Sham group) as well as of sarcoma 180-bearing animals treated with PBS (negative control, *per os*), methotrexate (MTX, 1.5 mg/kg, i.p.), or SE (50, 100 and 200 mg/kg, *per os*).

Parameter	Treatment					
	Sham	Negative Control	MTX	SE (50 mg/kg)	SE (100 mg/kg)	SE (200 mg/kg)
Albumin (g/dL)	36.59 ± 2.32	36.74 ± 2.41	32.14 ± 4.12 *	35.07 ± 3.02	35.15 ± 3.27	36.70 ± 0.44
Alanine aminotransferase (U/L)	67.51 ± 3.36	67.28 ± 4.35	102.52 ± 9.78 *	68.42 ± 5.15	66.25 ± 4.38	66.62 ± 5.48
Aspartate aminotransferase (U/L)	95.43 ± 6.14	94.43 ± 5.59	155.79 ± 12.51 *	94.14 ± 7.33	90.44 ± 8.12	92.85 ± 5.04
Alkaline phosphatase (U/L)	13.86 ± 0.71	14.26 ± 0.60	17.43 ± 0.80 *	14.01 ± 0.70	14.09 ± 0.86	14.09 ± 0.32
Bilirubin (mg/dL)	0.73 ± 0.19	0.59 ± 0.15	0.65 ± 0.18	0.57 ± 0.14	0.69 ± 0.23	0.42 ± 0.41
Gamma-glutamyl transferase (U/L)	14.95 ± 0.93	15.75 ± 0.84	15.33 ± 0.95	15.49 ± 1.02	15.56 ± 1.09	14.14 ± 0.53
Total protein (g/dL)	75.42 ± 6.42	75.23 ± 6.41	51.54 ± 5.45 *	73.06 ± 5.18	74.60 ± 5.15	75.46 ± 5.35
Blood urea (mg/dL)	0.32 ± 0.07	0.29 ± 0.06	0.65 ± 0.08 *	0.37 ± 0.08	0.31 ± 0.09	0.48 ± 0.08
Creatinine (mg/dL)	0.47 ± 0.09	0.42 ± 0.04	2.04 ± 0.40 *	0.49 ± 0.07	0.52 ± 0.08	0.44 ± 0.15
Total cholesterol (mg/dL)	96.58 ± 7.45	96.77 ± 5.67	90.55 ± 6.13	94.33 ± 6.15	85.35 ± 5.16 *	77.49 ± 7.54 *
Triglycerides (mg/dL)	99.27 ± 10.31	95.51 ± 9.69	91.42 ± 8.10	88.13 ± 9.13	80.22 ± 9.18 *	74.88 ± 6.65 *

Values represent the mean ± SEM of data from six animals (*n* = 6/group). * *p* < 0.05 indicates significant differences in relation to the Sham group evaluated by one-way ANOVA followed by Tukey’s test.

## Data Availability

Data are contained within the article.
